# Exploiting Bacteria for Improving Hypoxemia of COVID-19 Patients

**DOI:** 10.3390/biomedicines10081851

**Published:** 2022-08-01

**Authors:** Vito Trinchieri, Massimiliano Marazzato, Giancarlo Ceccarelli, Francesca Lombardi, Alessandra Piccirilli, Letizia Santinelli, Luca Maddaloni, Paolo Vassalini, Claudio Maria Mastroianni, Gabriella d’Ettorre

**Affiliations:** 1 Department of Public Health and Infectious Diseases Sapienza, University of Rome, 00185 Rome, Italy; vito.trinchieri@uniroma1.it (V.T.); massimiliano.marazzato@uniroma1.it (M.M.); letizia.santinelli@uniroma1.it (L.S.); luca.maddaloni@uniroma1.it (L.M.); paolo.vassilini@uniroma1.it (P.V.); claudio.mastroianni@uniroma1.it (C.M.M.); gabriella.dettorre@uniroma1.it (G.d.); 2 Department of Life, Health & Environmental Sciences, University of L’Aquila, 67100 L’Aquila, Italy; francesca.lombardi@univaq.it; 3 Department of Biotechnological and Applied Clinical Sciences, University of L’Aquila, 67100 L’Aquila, Italy; alessandra.piccirilli@univaq.it

**Keywords:** oxygen, CPAP, probiotics, SLAB51

## Abstract

Background: Although useful in the time-race against COVID-19, CPAP cannot provide oxygen over the physiological limits imposed by severe pulmonary impairments. In previous studies, we reported that the administration of the SLAB51 probiotics reduced risk of developing respiratory failure in severe COVID-19 patients through the activation of oxygen sparing mechanisms providing additional oxygen to organs critical for survival. Methods: This “real life” study is a retrospective analysis of SARS-CoV-2 infected patients with hypoxaemic acute respiratory failure secondary to COVID-19 pneumonia undergoing CPAP treatment. A group of patients managed with ad interim routinely used therapy (RUT) were compared to a second group treated with RUT associated with SLAB51 oral bacteriotherapy (OB). Results: At baseline, patients receiving SLAB51 showed significantly lower blood oxygenation than controls. An opposite condition was observed after 3 days of treatment, despite the significantly reduced amount of oxygen received by patients taking SLAB51. At 7 days, a lower prevalence of COVID-19 patients needing CPAP in the group taking probiotics was observed. The administration of SLAB51 is a complementary approach for ameliorating oxygenation conditions at the systemic level. Conclusion: This study proves that probiotic administration results in an additional boost in alleviating hypoxic conditions, permitting to limit on the use of CPAP and its contraindications.

## 1. Background

Acute Respiratory Distress Syndrome (ARDS) ensues in patients with serious lung diseases, including severe acute respiratory syndrome coronavirus type 2 (SARS-CoV-2). It is characterized by loss of alveolar-capillary barrier integrity and the formation of pulmonary edema manifested clinically by reduced oxygenation, decreased lung compliance, and bilateral radiographs infiltrates on chest radiographs. The lung is in such a condition that it takes time for the damage to resolve spontaneously. The task of the doctors is to keep the organs essential for survival, i.e., brain, kidneys, and liver, adequately oxygenated and thus functional [1].

The standard treatment for ARDS is mechanical ventilation (MV), but it is not devoid of risks to the patients. The physical forces generated during MV may cause a phenomenon called Ventilator-Induced Lung Injury (VILI). MV damage can occur for several reasons, e.g., excessive dilatation due to high blood flow, increased airway pressure, and inadequate positive expiratory closure pressure (PEEP) responsible for the alveoli collapse and reopening. These forces may damage the alveolar-capillary barrier and cause the release of mediators and the mobilization of inflammatory cells that further injure the barrier. Therefore, trying to keep the patients oxygenated and at the same time avoid MV or reduce its duration is one of the problems doctors have to deal with daily [2].

Okabe and colleagues recently reported intestinal ventilation in preclinical mammalian models, which may provide a novel route of oxygen delivery for patients requiring immediate respiratory support [3]. In the experimental model, to establish accessory respiration mechanisms in organs other than the lung, an intra-rectal oxygen gas O_2_ ventilation (g-EVA) or liquid ventilation (l-EVA) with oxygenated perfluorocarbon, were used. At that scope, they abraded the mucosal barrier to achieve a thinning of epithelial layers to facilitate gas exchange via the distal gut. The approach proposed by Okabe is fascinating because it provides an amount of oxygen through the colon in addition to that obtained through the lungs or MV. Unfortunately, there is the need for abrasion of the colonic mucosal barrier to facilitate the gas exchange, which may pose additional risks to the patients and require an intra-rectal administration of chemicals.

We propose a different approach to combat the hypoxia of ARDS patients, and it is based on the “saving of oxygen” in the upper gut. The intestinal epithelium of adult humans forms a multi-layered cell layer with a surface area of approximately 250–300 m^2^. Beneath the epithelium is a network of blood and lymph vessels essential for transporting nutrients and oxygen to the tissue. Microbiota also influences the oxygen levels of the gut lumen and of intestinal villi. SLAB51 formulation is a blend of probiotic bacteria endowed with arginine deiminase activity (ADI) [4]. ADI inhibits the arginine-dependent synthesis of nitric oxide (NO). NO is known to alter hypoxic sensing being able to induce hypoxia-inducible factor-1α (HIF-1α) destabilization by directly or indirectly activating prolyl hydroxylases (PHD) [5]. NO is also found to antagonize iron chelator–induced HIF-1α accumulation, which was ascribed to increases in intracellular free iron [5,6]. Previously, we have supplemented SLAB51 to sixty-nine severe COVID-19 patients requiring non-invasive oxygen therapy and presenting a CT lung involvement ≥ 50%. Patients receiving SLAB51 in addition to the standard therapy needed significantly lower oxygen amounts during the 24 h observation period. Furthermore, they presented higher blood levels of pO_2_, O_2_Hb, and SaO_2_ than the control group [7]. Our conclusion was that the administration of SLAB51 activates an “oxygen sparing effect” in the upper gut, not related to the colonic colonization by SLAB51 but due, al least partly to the inhibition of nitric oxide synthesis and the consequent impact on HIF-1α pathway. In this regard, it seems interesting to cite the recent report by Bonfili and colleagues showing that, in the Alzheimer’s disease mouse model, the oral administration with SLAB51 induced the up-regulation in the brain tissue of HIF-1α expression, which in turn was associated with a significant reduction in the expression of inducible nitric oxide synthase (NOS-2) [8].

To further extend the knowledge, we have conducted the present study on COVID-19 patients who required machines providing a continuous flow of air at a constant pressure of oxygen (CPAP; Continuous Positive Airway Pressure) for their hypoxia. Our results confirm that the probiotic SLAB51 can significantly reduce the duration of the CPAP treatment, with all that this implies about decreased risks for VILI and ease of patient management.

## 2. Methods

### 2.1. Design of the Study, Population, Settings and Data Collection

This “real life” study is a retrospective analysis of SARS-CoV-2 infected patients with hypoxaemic acute respiratory failure (hARF) secondary to COVID-19 pneumonia undergoing helmet Continuous Positive Airway Pressure (CPAP) treatment. Patients hospitalized between November 2020 and March 2021 in Policlinico Umberto I, University “Sapienza”, Rome, Italy and meeting the inclusion/exclusion criteria were retrospectively recruited.

Inclusion criteria were patients > 18 years old, with diagnosis of COVID-19, a CT scan confirming diagnosis of SARS-CoV-2 related pneumonia, and the need to start the CPAP support in the first 24 h after the admission to the hospital. Exclusion criteria were pregnancy, other concomitant chronic or acute lung diseases, contraindications to CPAP support (need for immediate intubation, Glasgow Coma Scale < 15; respiratory acidosis, systolic blood pressure (SBP) < 90 mmHg despite fluid resuscitation and/or use of vasopressors, swallowing disturbance with increasing risk of aspiration pneumonia, and inability to protect the airways).

**Figure 1:**
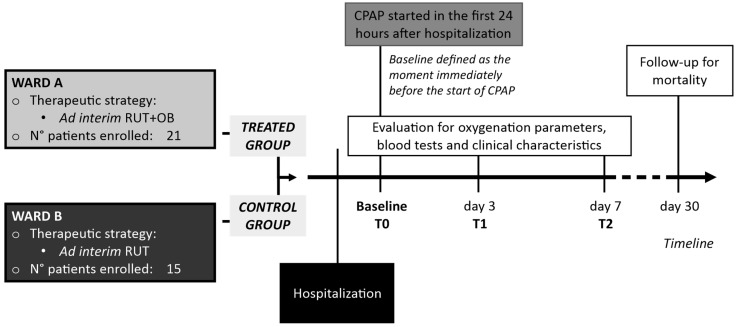
Schematic representation of the study design.

The patients progressively included in the study were hosted in two different wards devoted to the management of COVID-19: in the first ward, a therapeutic strategy including ad interim routinely used therapy (RUT) was adopted, while in the second one, RUT associated with SLAB51 oral bacteriotherapy (OB) supplementation was administered (treated group). The healthcare personnel of the two wards had similar expertise in the management of COVID-19 and a similar level of severity of managed patients. All of the patients were evaluated at baseline (T0), at day 3 (T1), and at day 7 (T2) for the oxygenation parameters, blood tests, and clinical characteristics. At 1 month of follow-up, COVID-19 related mortality was checked. The baseline was defined as the moment immediately before the start of CPAP support (Figure 1).

The source for patient data was medical records stored in the Electronic Information System of the wards involved: the variables considered included: (1) anamnestic data, (2) past clinical history (comorbidities), (3) current clinical history, treatments, (4) Arterial blood gas analysis, vital signs, and laboratory data. The study’s primary endpoint was to evaluate the timing spent in CPAP measured in days, the secondary endpoint was the mortality at 30 days.

### 2.2. Diagnosis of SARS-CoV-2 Infection, Radiological Staging and Treatment

The COVID-19 case definitions of *European Centre for Disease Prevention and Control* (ECDC) were adopted [8]. A suspected COVID-19 diagnosis was confirmed if SARS-CoV-2 nucleic acid was detected by reverse transcriptase-polymerase chain reaction (RT-PCR) in a clinical specimen. Stratification of COVID-19 severity was based on *World Health Organization* (WHO) criteria [9].

All of the patients underwent high-resolution CT/non-contrast enhanced chest CT at the admission in hospital. Multidetector CT scanners (Somatom Sensation 16 and Somatom Sensation 64; Siemens Healthineers) were used for all examinations. As previously reported, a semi-quantitative CT score was adopted to calculate the extent of anatomic involvement, as follows: 0, no involvement; 1, <5% involvement; 2, 5–25% involvement; 3, 26–50% involvement; 4, 51–75% involvement; and 5, >75% involvement. The sum of each individual lobar score gave the resulting global CT score (0 to 25).

The patients were treated with ad interim therapy as suggested by the Italian Medicine Agency (AIFA) and the Italian Society of Infectious and Tropical Diseases (SIMIT) [10]. In detail: remdesivir + dexamethasone (6 mg daily for 10 days) plus low molecular weight heparin (prophylactic/therapeutic dosage) ± eventually antibiotic treatment.

Oral bacteriotherapy was administered in 3 equal doses daily for a total of 2400 billion bacteria per day. The administered SLAB51 multi-strain probiotic formula was composed by *Streptococcus thermophilus* DSM32245, *Bifidobacterium animalis* subsp. *lactis* DSM32246, *Bifidobacterium animalis* subsp. *lactis* DSM 32247, *Lactobacillus acidophilus* DSM 32241, *Lactobacillus helveticus* DSM 32242, *Lactobacillus paracasei* DSM 32243, *Lactobacillus plantarum* DSM 32244, and *Lactobacillus brevis* DSM 27961 (Sivomixx800^®^, Ormendes SA, Lausanne, Switzerland).

All patients included in the study were supported by oxygen therapy delivered via the use of helmet CPAP. The treatment was started in the first 24 h after hospitalization. Helmet CPAP was considered in patients with arterial oxygen pressure (PaO_2_) < 60 mmHg on arterial blood gas analysis and/or respiratory rate (RR) > 30/min after being on maximal oxygenation therapy by 15 lpm Venturi mask for 15 min. Criteria for intubation were: persistent or worsening of acute respiratory failure (SpO_2_ < 88%, RR > 30/min) despite CPAP set to FiO_2_ 100% and PEEP 10 cmH_2_O [11].

Contraindications to CPAP support were reported in the exclusion criteria. Patients who failed CPAP therapy underwent invasive mechanical ventilation (IMV); IMV was considered after 4 days of unsuccessful treatment with CPAP support [12].

### 2.3. Malondialdehyde (MDA) Level Assay

The plasma levels of malondialdehyde were measured at the application of CPAP and after 3 days from the beginning of treatments by using MDA ELISA kit (Elabscience, Houston, TX, USA), according to the manufacturer’s instructions. The absorbance was measured by spectrophotometric reading at 450 nm using a microplate reader (Bio-Rad, Hercules, CA, USA).

### 2.4. Ethics Committee Approval

The Ethics Committee of University of Rome Sapienza (Italy)/Azienda Ospedaliero-Universitaria Policlinico Umberto I of Rome (Italy) approved the study with number 109/2020. Reporting of the study conforms to broad EQUATOR guidelines.

### 2.5. Statistical Analysis

No sample size calculations were performed. The categorical variables were compared using the χ^2^ test with Yates’ continuity correction and shown as absolute frequencies and percentages. The bilateral Mann-Whitney U test was used for continuous variables to determine statistically significant differences between groups at each considered time point while, for each group, the Wilcoxon signed-rank test was used to assess significant differences between consecutive time points. In each case, a *p*-value ≤ 0.05 was considered statistically significant. Analyses were performed by using the statistical software R 4.0.3 (R Core Team, Vienna, Austria).

## 3. Results

**Figure 2:**
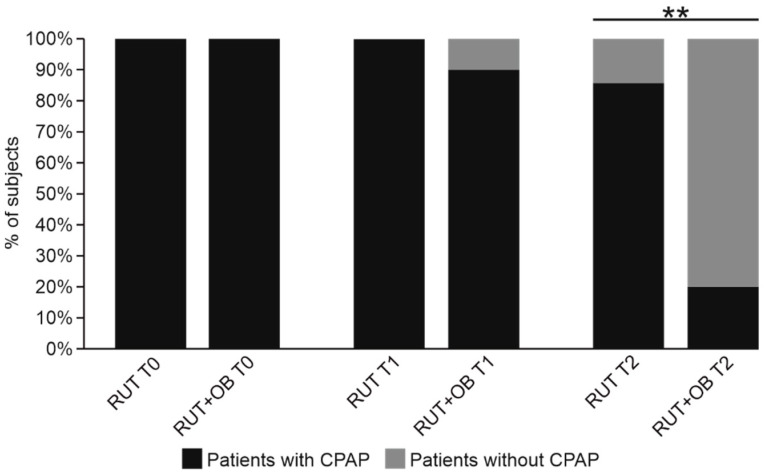
Distribution of patients with CPAP between groups at different time points. Data are represented as boxplots showing median, IQR (25–75%). **: *p* ≤ 0.001.

We performed the analysis on a total of 36 subjects in need of CPAP, of which 21 (58.3%) were also treated with SLAB51, while 15 (41.7%) constituted the control group. (Figure 2).

No fatal events were observed in the two groups during the observation period (T0–T2). At 30 days from admission to the hospital, although not statistically significant, the RUT group presented a higher prevalence of fatal events than the RUT+OB one (RUT 3/14(21.4%) vs. RUT+OB 1/21(4.8%), *p* = 0.3219). The two groups were homogenous with respect to the demographic and clinical variables reported in Table 1.

Before the beginning of the observation period, all of the patients had been treated for about 24 h with continuous CPAP. Concerning the prevalence of subjects needing CPAP, the two groups were still comparable at T1 (Controls 100% vs. Treated 90% *p* = 0.501) while at T2, a significantly lower proportion of patients in the RUT+OB group were in need of CPAP, compared to the RUT one (RUT 12/14, 85.7% vs. RUT+OB 4/21 (19%) *p* = 0.0003) (Figure 2).

**Figure 3:**
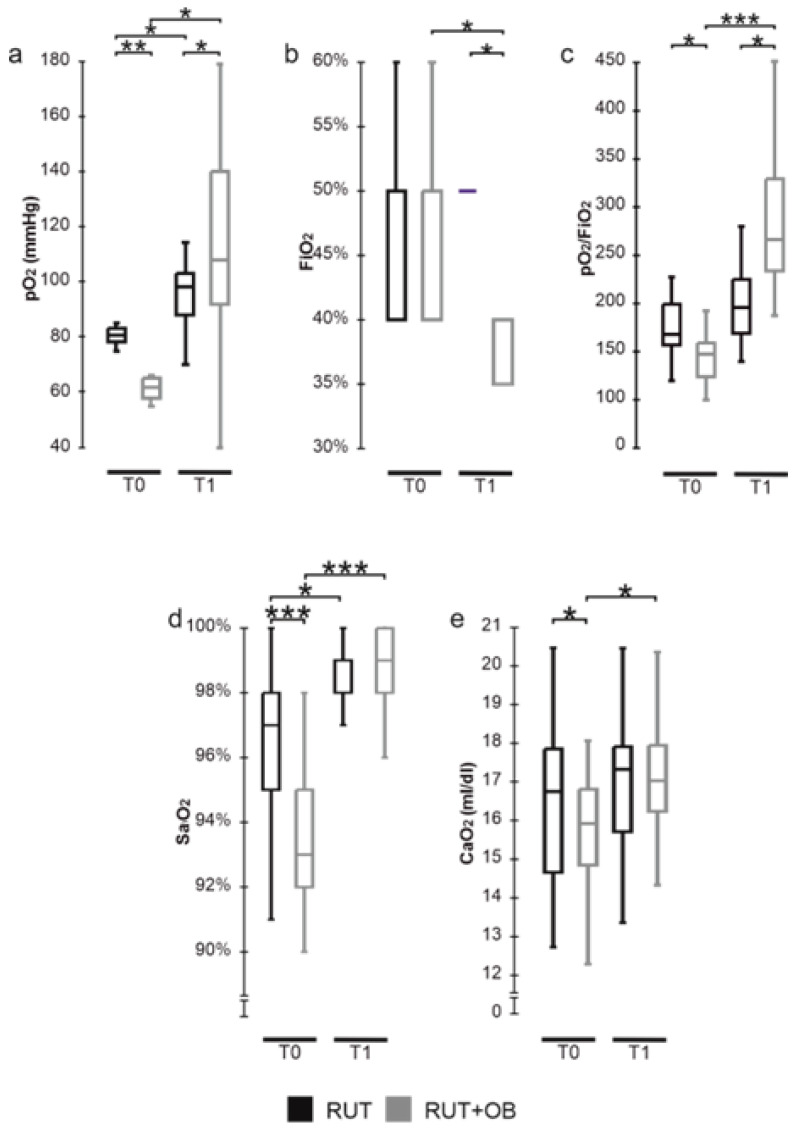
Distribution of blood oxygenation variables between groups at different time points: (**a**) pO_2_ (mmHg); (**b**) FiO_2_; (**c**) pO_2_/FiO_2_;(**d**) SaO_2_; (**e**) CaO_2_ (mL/dL). Data are represented as boxplots showing median and IQR (25–75%). *: *p* ≤ 0.05; **: *p* ≤ 0.001; *** *p* < 0.0001.

Significantly lower values of pO_2_ were determined for the RUT+OB group with respect to controls at T0, while the situation was the opposite at T1 (Figure 3a). The FiO_2_ values recorded at T0 confirm that the two groups were in the same need for oxygen while at T1, the levels of FiO_2_ were significantly reduced in the patients treated with SLAB51 compared to the subjects of the RUT group (Figure 3b). In line with our observations for the pO_2_ and FiO_2_ levels, at T0, the treated group also presented significantly lower values of the pO_2_/FiO_2_ (P/F ratio) compared to the control one. At T1, the group administered with SLAB51 evidenced a significantly higher P/F with respect to controls (Figure 3c). Even though we registered significantly lower SaO_2_ levels in the treated group than in controls at T0, the two groups were homogeneous for this oxygenation parameter after three days (Figure 3d). Concerning the arterial oxygen content (CaO_2_), no difference was present between the two groups at T1, even though at T0, the treated group displayed significantly lower levels of CaO_2_ than the controls. (Figure 3e).

**Figure 4:**
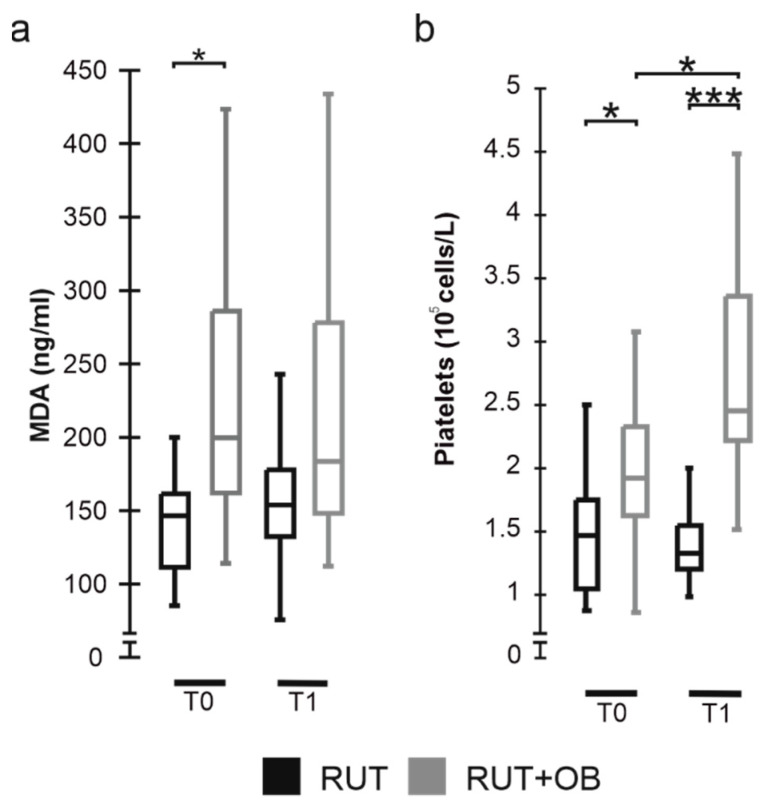
Distribution of MDA levels (**a**) and platelet counts (**b**) between groups at different time points. Data are represented as boxplots showing median and IQR (25–75%). *: *p* ≤ 0.05; *** *p* < 0.0001.

We have also evaluated MDA levels, which indicates the degree of oxidative stress in the blood. Results show that subjects treated with SLAB51 presented significantly higher blood MDA values at the beginning of the study than those treated with only the standard therapy. Although not significantly, the group treated with oral bacteriotherapy tended to reduce blood MDA over time, while an opposite behavior has been observed for the control group (Figure 4a).

All subjects considered in the study were characterized by thrombocytopenia (platelets < 150 × 10^9^ cells/L). At the beginning of the studied period, significantly higher counts were observed for the RUT+OB than for the RUT one. Significant increases in platelets counts were further observed for the treated group at T1, while an opposite condition was shown by controls (Figure 4b). Overall, results evidenced an opposite behavior for the two groups, with the RUT+OB subjects increasing the number of blood platelets and the control group tending to lower the levels of such cells.

## 4. Discussion

Associations between gut microbiota composition, levels of cytokines, and inflammatory markers in patients with COVID-19 suggest that several gut microbial components are involved in the magnitude of COVID-19 severity, possibly via modulating host immune responses [13]. Based on this premise, a series of studies are in progress to evaluate the impact of probiotics on subjects with COVID-19. The presumed beneficial effect of probiotic bacteria in individuals infected with SARS-CoV-2 would be based on gut colonization by anti-inflammatory strains, production of lactic acid and direct anti-viral molecules, breakdown of polyphenols into more bioactive varieties able to reduce systemic inflammation, increased vitamin D absorption and bioactivity, enhancement of intracellular oxidative enzyme and upregulation of antioxidant enzymes, fermentation of otherwise poorly digestible dietary carbohydrates into Short-Chain Fatty Acids (SCFA) which are a source of energy for gut cells and improve gut wall integrity [13,14].

Many other mechanisms can be added to the above list. However, it should be noted that whatever the mechanism/s underlying the above suggested protective actions of probiotics would be their primary site of action is the colon; moreover, they are all characterized by the fact that to be set in motion and effective, they require a continuous and prolonged administration. Two recent studies confirm this “time factor”. A randomized, placebo-controlled, double-blind trial has shown that *Loigolactobacillus coryniformis* K8 may improve the COVID-19 vaccine-specific responses in elderly populations when the product is administered for 21 days [15]. In another paper, *Lactobacillus rhamnosus* GG (LGG) was able to protect against COVID-19 symptoms development when used as post-exposure prophylaxis for 28 days [16]. The probiotic administration was intended as a prophylactic measure and not as a treatment in both trials.

According to our clinical experience, the risk of developing respiratory failure for COVID-19 patients is reduced eightfold by administering, in addition to the routinely used therapy, a specific formulation of probiotic bacteria, SLAB51 [17]. A retrospective study on 200 patients confirmed that subjects treated with SLAB51 had a significantly higher chance of survival [18].

The present study reinforces our previous data [6,17,18,19]. We recently presented evidence that SLAB51 possesses high levels of ADI [6]. This predominantly prokaryotic enzyme pathway catalyzes the conversion of arginine to ornithine, ammonium, and carbon dioxide while generating ATP from ADP and phosphate [20]. Using the same substrate as NOS, L-arginine, to which ADI has a high affinity [21], the bacterial enzyme indirectly inhibits the generation of NO due to substrate depletion [21,22]. Of note, the exposure of human intestinal epithelial cells Caco-2 to SLAB51 induced a reduction in NOS_2_ activity [6]. Enzymatic NO formation catalyzed by NOS comprises a series of redox reactions, with degradation of L-arginine to L-citrulline and NO. The reaction consumes 1.5 mol of NADPH and 2 mol of O_2_ per mol of L-citrulline formed. Moreover, NO has a short half-life being highly reactive and rapidly scavenged by endogenous compounds, including O_2_ [23,24]. Considering that under normal conditions, the intestinal mucosa receives between 10% and 35% of the total cardiac output [25], we speculated that the administration of SLAB51, inhibiting the NOS_2_ activity in the gut and consequently the oxygen consumption by the relative enzymatic reaction, could increase the systemic O_2_ availability.

We supplemented the two groups with identical amounts of oxygen, monitored its administration continuously, and adjusted its level according to the patient’s clinical condition. From T0 to T2, the progressive amelioration of hypoxia permitted a concomitant reduction in the quantity of oxygen administered to the patients treated with SLAB51. At T2, a significantly lower proportion of patients taking SLAB51 were still in need of CPAP compared to the controls.

A surprising finding observed during the SLAB51 administration is its influence on platelet count and oxidative stress status. Thrombocytopenia is a hallmark of severe COVID-19 infections associated with abnormal coagulation function and increased mortality [26,27]. Low platelet count can be attributed to platelet apoptosis, and hyperactivation leading to the incorporation of platelets into microthrombi responsible for the severe thrombotic events observed in COVID-19 patients [28,29]. Hypoxia plays a role in the hyperactivation of platelets and excessive thrombin formation during COVID-19 [26,27,28,29,30]. The prothrombotic effect of hypoxia is driven, almost in part, by the alteration of the homeostasis of hypoxia-inducible factors (HIFs) in platelets [31]. HIFs consist of an oxygen sensing system expressed in various human cell types, and responsible for adaptation to hypoxia. Recently, an in vivo study performed by Bonfili and colleagues evidenced that the oral administration of SLAB51 is associated with modifications in the homeostasis of HIFs, particularly that of the HIF-1α subunit [7]. Although in this study we did not provide direct evidence about the mechanism(s) associating the SLAB51 administration and the increased amount of blood circulating platelets, we can hypothesize that the significantly increased platelet count observed for subjects under probiotic administration could directly reflect a reduced activation of platelets due to the alleviation of blood hypoxia, and the modulation of HIFs.

In patients supplemented with SLAB5, we also observed a progressive lowering of the blood levels of MDA, one of the most critical end products of lipid peroxidation that is often used as a biomarker in determining oxidative stress [32,33].

In the acute course of the COVID-19 disease, redox biomarkers correlate with most inflammatory and multiorgan impairment biomarkers. They are associated with changes in the chest multidetector computed tomography findings in patients with COVID-19 pneumonia [34].

CPAP is a form of respiratory support that can help patients with respiratory distress and increased oxygen requirements. The objective of CPAP treatment is to augment functional residual capacity, improve oxygenation, increase lung compliance, and possibly avoid intubation to maintain acceptable oxygenation [35]. That CPAP has significant contraindications is well known, and therefore, any solution to reduce CPAP duration is desirable [36,37]. We compared patients treated with CPAP and RUT to patients also supplemented with SLAB51 to verify the effect of multiple administrations of SLAB51 in a well-controlled environment in terms of oxygen supplementation. As far as we know, no medical therapeutic approaches can further improve the oxygenation of the COVID-19 patient treated with CPAP without significant changes in lung imaging and function.

The lack of randomization, the absence of a placebo, and the limited number of patients represent the limits of the study but do not invalidate the importance of obtained results. While waiting for the currently available therapies to run their course and the lung to regain full function, SLAB51 supplementation is a safe and cheap approach for alleviating hypoxia at the systemic level and then in the organs essential for the maintenance of the life of the patient. The specific action exerted by the SLAB51 multi-strain probiotic should be framed in the modulation of agonists and antagonists of oxygenation in the small intestine.

## Figures and Tables

**Table 1:** Distribution of demographic and clinical variables between groups. A *p*-value ≤ 0.05 was considered statistically significant.

	RUT (No. 21) Median(IQR) or No.(%)	RUT+OB (No. 15) Median(IQR) or No.(%)	*p*-Value
Gender (Female)	4 (28.6)	13 (65)	0.08
Age (Years)	66 (60–68)	64 (54–73)	0.74
D-dimer µg/L	667.5 (379.7–1332)	749 (447–1094)	0.94
Fibrinogen g/L	4.5 (3.6–5.1)	4.4 (3.7–5.1)	0.92
Protein C reactive PCR µg/L	14,500 (1300–52,550)	28,000 (12,200–53,200)	0.51
High Flux Oxygen (%)	14/14 (100)	21/21 (100)	1

## Data Availability

All data is contained within the article.
